# Regulating the Secondary Use of Data for Research: Arguments Against Genetic Exceptionalism

**DOI:** 10.3389/fgene.2019.01254

**Published:** 2019-12-20

**Authors:** Andrea Martani, Lester Darryl Geneviève, Christiane Pauli-Magnus, Stuart McLennan, Bernice Simone Elger

**Affiliations:** ^1^ Institute for Biomedical Ethics, University of Basel, Basel, Switzerland; ^2^ Department of Clinical Research, University and University Hospital of Basel, Basel, Switzerland; ^3^ Institute of History and Ethics in Medicine, Technical University of Munich, Munich, Germany; ^4^ University Center of Legal Medicine, University of Geneva, Geneva, Switzerland

**Keywords:** secondary use, research policy, genetic data, genetic exceptionalism, data protection

## Abstract

As accessing, collecting, and storing personal information become increasingly easier, the secondary use of data has the potential to make healthcare research more cost and time effective. The widespread reuse of data, however, raises important ethical and policy issues, especially because of the sensitive nature of genetic and health-related information. Regulation is thus crucial to determine the conditions upon which data can be reused. In this respect, the question emerges whether it is appropriate to endorse genetic exceptionalism and grant genetic data an exceptional status with respect to secondary use requirements. Using Swiss law as a case study, it is argued that genetic exceptionalism in secondary use regulation is not justified for three reasons. First, although genetic data have particular features, also other non-genetic data can be extremely sensitive. Second, having different regulatory requirements depending on the nature of data hinders the creation of comprehensible consent forms. Third, empirical evidence about public preferences concerning data reuse suggests that exceptional protection for genetic data alone is not justified. In this sense, it is claimed that regulation concerning data reuse should treat genetic data as important, but not exceptional.

## Introduction

The considerable potential that the extensive use of data in the medical field can disclose has been extensively discussed (Costa, 2014). Not only can data be exploited at an individual level to accurately implement personalised medicine (Meier-Abt et al., 2018)^1^, but it can also be extremely useful at a societal level to help develop cost-efficient healthcare policies and carry out clinical and public health research (Dugas et al., 2013).

Yet, alongside with many expected beneficial impacts, the full deployment of data in the healthcare sector also raises challenging legal and ethical questions. A great deal of these is related to the high mobility and interconnectivity of information which the big data era has brought about (Mittelstadt and Floridi, 2016). In a context where technical advances make it possible for data to be stored for a long time and to move quickly and unrestrained, data can be easily shared and subsequently reused. As a consequence, the distance increases between subjects and their personal information (Nuffield Council on Bioethics, 2015). It then becomes crucial to combine the pervasive and beneficial use of data with efficacious safeguards capable of protecting sensitive personal data, such as genetic and non-genetic health data (Jensen et al., 2012).

Finding the right balance between protecting privacy and promoting beneficial use of data is particularly difficult in the case of secondary use. In contrast to primary use, where data are collected and then used for a specific aim, secondary use entails the processing of data for different purposes to those originally envisaged when information is gathered and, potentially, also the involvement of data processors other than the primary data collectors (Schlegel and Ficheur, 2017). Conducting multiple secondary analyses on the same data has the potential to reduce costs and time for research (Safran et al., 2007; Geissbuhler et al., 2013). If usage of data was limited to primary purposes, subjects’ integrity and privacy would be invaded more often, as data would have to be collected from them for every single data-usage. Moreover, data collection and analysis would become lengthier and more expensive (The Danish Council of Ethics, 2015), since new datasets would have to be created every time a new aim emerges.

Secondary use of data is important because of the many purposes for which data can be reused in the healthcare sector. These include organizational, educational, public health, commercial, disease surveillance, quality measurement, and forensic purposes (Safran et al., 2007; Elkin et al., 2010; Barton et al., 2011). For example, digitalized histology slides can be used to train pathologists and routinely collected data from hospitals can be used for quality improvement and biomedical research. Moreover, the fact that data do not have a strong tangible and physical dimension entails that multiple secondary uses of data are not mutually excluding. While re-using tissues or biological material is usually^2^ possible for a finite amount of times, iterative access and exploitation of the same data unit do not affect the integrity of either the single piece of information or the entire dataset where it belongs. As long as the single piece of information is not erased or lost, the same data can be cumulatively used for research, public health, clinical, and commercial purposes, thus generating an incentive to rely on information which has already been collected (Richter et al., 2016).

Data reuse can be also be beneficial as many health systems are promoting the idea of learning healthcare, which has been described as the attempt to “generate and apply the best evidence for the collaborative healthcare choices of each patient and provider; to drive the process of discovery as a natural outgrowth of patient care; and to ensure innovation, quality, safety, and value in health care” (Institute of Medicine, 2007:37). In the framework of learning healthcare, reuse of data collected in the clinical setting is fundamental, as it allows to conduct a wide range of healthcare research projects, whose results can then be “*fed-back*” to the healthcare system to improve the delivery and quality of care (Budrionis and Bellika, 2016). In fact, a core component of learning healthcare is to repeatedly exploit data routinely collected at different points of the care-cycle in multiple forms—such as electronic health records, health registries or laboratory tests—to fuel the chain of healthcare improvements (Deeny and Steventon, 2015; Meystre et al., 2017).

In a recent review of projects involving the secondary use of data, Martin-Sanchez et al. (2017) identified three main categories of cutting-edge initiatives in this field. Firstly, there are projects reusing data for clinical research, where patient data previously collected at different steps of their clinical management can accelerate recruitment and reduce redundant data capture. Secondly, data are increasingly reused for different types of evaluations of health interventions, in which routine data of patients undergoing alternative treatments can be used to retrospectively compare them. Thirdly, many projects have started reusing data in the field of genomic research and research concerning the effects of the environment on health. The latter category is particularly innovative, since these kind of projects often combine the reuse of both genetic and other health related medical information. For example, the eMerge Network initiative in the United States aims at linking genetic data from multiple biorepositories with other clinical health data, which would allow to study the association of genome-wide data with phenotypes defined through the electronic medical records data (McCarty et al., 2011).

Within this context, the objective of this paper is to discuss whether granting genetic data a special status in the regulation of data reuse represents a justified policy choice. To answer such a question, this contribution delineates the prevalent regulatory frameworks at both national and international levels and then compares them with Swiss law, which represents a rare case where genetic data are given an exceptionally special status with respect to secondary use requirements. The analysis of this unique normative framework is complemented by policy considerations, whereby the problematic aspects of endorsing genetic exceptionalism in regulation concerning the reuse of data are underscored. It is finally argued that the case of Switzerland suggests that granting genetic data an exceptionally special status in terms of reuse requirements is not an appropriate policy choice.

## Secondary Use of Data for Research Purposes: Switzerland’s Unique Regulatory Framework

A supportive policy environment has been described as one of the crucial elements to favor the reuse of data (Safran et al., 2007). This entails having a regulatory framework that facilitates secondary use, but also protects privacy and autonomy (Sethi and Laurie, 2013). In order to strike a balance between these two elements, regulations normally establish that research involving the secondary use of personal health information can be permitted only if consent has been obtained from data subjects, the law and/or a research ethics committee (REC) has granted an exemption or the data have been anonymized or de-identified (Lowrance, 2003). This is the case, for example, for regulation and guidelines covering the processing of data for research in the EU, the US, and in Canada (**Table 1**).

**Table 1:** Requirements for the secondary use of data for research purposes.

	Informed consent	Research exemption	Data anonymization/de-identification^a^
European Union	The use (primary or secondary) of all sensitive types of data is permitted if subjects consent. [General Data Protection Regulation (GDPR), 2018, art. 9.2(a)].	The use (primary or secondary) of all sensitive types of data is permitted if the research exemption applies. [GDPR, 2018, art. 9.2(j)].^3^	The use (primary or secondary) of all types of data for research is permitted if the data are anonymous or anonymized. (GDPR, 2018, recital 26).
United States	Any use of protected health information (primary or secondary) cannot be done without the explicit authorization by data subjects [Health Insurance Portability and Accountability Act of 1996 (HIPAA) (2013), §164.508].	An Institutional Review Board (IRB) or a privacy board can allow for research involving the use (primary or secondary) of protected health information to be conducted without data subjects’ authorization [HIPAA (2013), §164.512].	The requirements for the use (primary or secondary) of personal health information do not apply if data have been de-identified [HIPAA (2013), §164.502].
Canada	Research involving secondary use of identifiable information requires data subjects’ consent. [Tri-Council Policy Statement 2 (TCPS 2), 2018, art. 5.5A].	Research involving secondary use of identifiable information can exceptionally be conducted without consent by authorization of a Research Ethics Board. (TCPS 2, 2018, art. 5.5A).	Researchers do not need to ask for consent if research relies exclusively on the secondary use of non-identifiable health. (TCPS 2, 2018, art. 5.5B).

^a^Definitions of anonymization or de-identification vary in different legislations (Elger and Caplan, 2006) and sometimes even within the same country.

In the framework of data processing, a hierarchy is usually established between sensitive and non-sensitive personal information. Sensitive data are granted a higher level of protection than other personal information, generally by limiting their processing—both primary and secondary—or by setting more stringent conditions for the usage or collection of such data. For example in the EU, the recent General Data Protection Regulation (GDPR) (2018) recognizes the special nature of some types of personal information and establishes a series of specific rules that must be complied with when such sensitive data are handled (Shabani and Borry, 2018).

Within this hierarchy, it is usually acknowledged that genetic and health-related information are part of that sensitive data requiring a higher level of protection, but it is not considered necessary to draw a significant distinction between genetic and non-genetic data in terms of reuse requirements. With respect to secondary use requirements in the field of research, it is common to consider genetic and other non-genetic health data as equally sensitive, and genetic data are not granted any exceptional status (Wilkinson, 2010; Kim et al., 2018).

In contrast, Swiss law sets unequal normative standards for data reuse depending on whether data are genetic or health-related. The secondary use of data for research purposes is regulated by the Human Research Act (HRA) (2014) and the Human Research Ordinance (HRO) (2014), two comprehensive pieces of law passed in 2014 at the federal level. The HRA and HRO exclusively regulate the field of biomedical research and entail a set of sector-specific rules for the processing of data in this field. From a legal point of view, these sector-specific rules function as *lex specialis*, i.e. they override the general data processing norms contained in the Federal and Cantonal data protection laws (Rütsche, 2015). The latter only have a *subsidiary* function with respect to the regulatory framework for the processing of data in the field of biomedical research set by the HRA and HRO. According to this sector-specific regulatory framework, the conditions to reuse genetic data for research purposes are stricter, whereas secondary use of non-genetic health data is subject to more relaxed legal requirements. With respect to secondary use for research, Swiss legislation follows the doctrine of genetic exceptionalism, i.e. the idea genetic information is uniquely personal and thus deserves special protection (Annas et al., 1995). Accordingly, reuse standards are different depending on the genetic or non-genetic nature of data (see **Table 2**).

**Table 2:** Requirements for legitimate secondary use of data in Switzerland.

	Secondary use of identified^a^ data	Secondary use of “coded”^a^ data	Anonymization^a^ of data for secondary use	Secondary use of anonymous^a^ information
Genetic data	Explicit consent must be obtained for every single research project [Human Research Act (HRA), 2014, art. 32.1].	Explicit consent is required, but it can cover multiple research projects (broad consent). (HRA, 2014, art. 32.2)	Explicit consent is NOT required, but data subjects have right to dissent (presumed consent). (HRA, 2014, art. 32.3)	No requirements.
Other health-related data	Explicit consent is required, but it can cover multiple research projects (broad consent). (HRA, 2014, art. 33.1)	Explicit consent is NOT required, but data subjects have right to dissent (presumed consent). (HRA, 2014, art. 33.2)	No requirements.	No requirements.

^a^The meaning of these terms in the Swiss context is clarified below in the corresponding paragraphs.

### The Definitions of Genetic and Non-Genetic Health Data

Similarly to other national and international regulations, Swiss law presents two different definitions for genetic and health data (**Table 3**).

**Table 3:** Definition of genetic and health-related data for the purpose of data processing: a comparison between different regulations.

	Definition of health-related data	Definition of genetic data
Switzerland	“*information concerning the health or disease of a specific or identifiable person, including genetic data.*” (HRA, 2014, art. 3.f).	“*information on a person’s genes, obtained by genetic testing*.” (HRA, 2014, art. 3.g).
European Union	“*personal data related to the physical or mental health of a natural person, including the provision of health care services, which reveal information about his or her health status*” [GDPR, 2018, art. 4(15)].	“*personal data relating to the inherited or acquired genetic characteristics of a natural person which give unique information about the physiology or the health of that natural person and which result, in particular, from an analysis of a biological sample from the natural person in question*” [GDPR, 2018, art. 4(13)].
United States	“*any information, including genetic information, whether oral or recorded in any form or medium, that:* *(1) Is created or received by a health care provider, health plan, public health authority, employer, life insurer, school or university, or health care clearinghouse; and* *(2) Relates to the past, present, or future physical or mental health or condition of an individual; the provision of health care to an individual; or the past, present, or future payment for the provision of health care to an individual.*” [HIPAA (2013), §160.103].	“*information about:* *(i) The individual’s genetic tests;* *(ii) The genetic tests of family members of the individual;* *(iii) The manifestation of a disease or disorder in family members of such individual; or* *(iv) Any request for, or receipt of, genetic services, or participation in clinical research which includes genetic services, by the individual or any family member of the individual.*” [HIPAA (2013), §160.103].
Canada	“*(a)information concerning the physical or mental health of the individual;* *(b)information concerning any health service provided to the individual;* *(c) information concerning the donation by the individual of any body part or any bodily substance of the individual or information derived from the testing or examination of a body part or bodily substance of the individual;* *(d) information that is collected in the course of providing health services to the individual; or* *(e) information that is collected incidentally to the provision of health services to the individual.*” [Personal Information Protection and Electronic Documents Act (PIPEDA), 2019, Part 1 Section 2].	There is no specific and uniform definition of genetic data (Walker, 2014).

According to the HRA (2014), health data include all pieces of “information concerning the health or disease of a specific or identifiable person, including genetic data” (HRA, 2014, Art. 3.f). To define genetic data Swiss regulation adopts a more pragmatic approach, if compared with its international counterparts. Unlike other regulations (**Table 3**) the Swiss definition covers “information of a person’s genes,” but only when this is “obtained by genetic testing” (HRA, 2014, art. 3.g). This implies that the special status granted to genetic data does not apply to all the genetic characteristics of a natural person, since, in order to qualify as “genetic” for the application of the law, data need both to satisfy a requirement about the nature of the information itself and about its source (Schweizer Bundesrat, 2009). In this sense, data concerning a person’s genes whose source is not a genetic test have to be considered as normal health data, as far as reuse requirements are concerned.

### Secondary Use of Identified Genetic and Non-Genetic Health Data

The distinctive feature of identified data is that it is so rich and comprehensive that it is possible to identify data subjects by looking at the single dataset alone and without the need to rely on any additional pieces of information (Heuberger-Götsch and Burkhalter, 2014). Since in this case data subjects can be easily tracked back, reuse requirements are generally strict.

For the secondary use of identified genetic data, Swiss law establishes that informed consent needs to be both specific and explicit (HRA, 2014, art. 32.1). In this case, consent must therefore be referred to clearly defined research project(s) and cannot cover broad or unspecified areas of research. Moreover, it must also be explicit, thus always requiring an affirmative action by the data subject, whose agreement cannot be presumed—for example—by using a consent form with pre-ticked boxes. Therefore, researchers willing to reuse identified genetic data need to re-contact all data subjects and obtain a renewed provision of consent for every new study involving their data. On the contrary, for non-genetic health data researchers still need to ask for explicit consent, but this does not need to be related to a specific study and can cover broad classes of research (HRA, 2014, art. 33.1 HRA). This type of consent is commonly referred to as “broad” or “general” (Grady et al., 2015) and offers the advantage of being valid for a wide range of research projects, even if these do not coincide with the initial reason for data collection (Petrini, 2010). Once data subjects have provided this form of consent, there is no need for researchers to re-contact participants before any new study involving the reuse of the same set of data, as long as research lies within the area that was covered by the initial provision of consent.

### Secondary Use of “Coded” Genetic and Non-Genetic Health Data

Secondary use requirements are different if personal data are “coded.” The key characteristic of “coded” data is that re-identification—although always possible—can only be achieved through the use of additional information to those present in the dataset, normally referred to as “key” or “code” (Heuberger-Götsch and Burkhalter, 2014). In the literature, a distinction is sometimes made between “coded” or “pseudonymized” data on the one hand and “reversibly anonymized” data on the other, depending on whether the key to re-identify data subjects is kept in-house by the researchers managing the dataset or is held by third parties (Elger and Caplan, 2006). Swiss definition of “coded” data, on the contrary, covers every personal information “linked to a specific person *via* a code” (HRA, 2014, art 3.h), whether or not researchers have direct access to the key necessary to re-identify data subjects. The law requires that the “key must be stored separately from the material or data collection [ … ] by a person to be designated in the application who is not involved in the research project” (HRO, 2014, art. 26.2). This resembles in part the requirements of the GDPR, which demands that the key “is kept separately and is subject to technical and organizational measures to ensure that the personal data are not attributed to an identified or identifiable natural person” [GDPR, 2018, art. 4(5)]. In this respect, it is also important to underscore that—according to Swiss regulation—as long as the key to achieve re-identification exists, data must be regarded as “coded” data and cannot be considered “anonymized” (Rütsche, 2015; McLennan et al., 2018).

In Switzerland, for “coded” genetic data, explicit consent must be obtained, either for a specific project or also for broad classes of research (HRA, 2014, art. 32.2). For “coded” non-genetic health data, on the contrary, explicit consent is not needed. If researchers provide some basic information to data subjects, consent can be implicitly presumed, as long as the data subjects have not explicitly dissented (HRA, 2014, art 33.2). Researchers simply have the duty to inform the individuals whose data are to be reused of the proposed use of the data, of the right to dissent, of the measures in place to protect information and of the possibility that data are passed over to third parties (HRO, 2014, Art. 32). With this form of “presumed” consent (also known as “*opt-out*” model), it remains a challenge to register an eventual dissent by the data subject (Rütsche, 2015; Swissethics, 2018). In this case, the *default option* (i.e. the case where the data subject does not explicitly neither consent nor dissent to the reuse of his data) is that secondary use of “coded” data is permissible, since consent is presumed.

### Secondary Use of Anonymized Genetic and Non-Genetic Health Data

As highlighted in **Table 1**, anonymization is commonly recognised by many regulations as a valid alternative to consent to reuse information without infringing on data subjects’ rights. This is due to the widespread regulatory assumption that anonymous data do not represent personal information (El Emam et al., 2015). This holds true also for Switzerland, where the lawmaker has established that anonymous data fall outside the scope of the HRA (HRA, 2014, art. 2.2), thus implicitly allowing to conduct research on non-identifiable data, regardless of the genetic or non-genetic nature.^4^

However, if genetic data are collected in an identifiable form and then only subsequently anonymized, special reuse requirements apply. According to the law, anonymization consists in the deletion of “all items which, when combined, would enable the data subject to be identified without disproportionate effort” (HRO, 2014, art. 25.2), including—in particular—metadata such as the “name, address, date of birth and unique identification numbers” (HRO, 2014, art. 25.2). If genetic data are anonymized following this process, secondary use for research purposes can be performed only if data subjects have not actively expressed their dissent (Swissethics, 2017) and if researchers fulfil some information duties. The latter include the obligation to inform data subjects of their right to dissent and of the possibility that data are transferred to third parties once anonymized (HRO, 2014, art. 30). Moreover, since advances in medical sciences have contributed to further enhance the predictive value of genetic data, the lawmaker also requires that subjects are warned that anonymization might entail indirect consequences on their state of health, since it might impede—for example—the return of clinically relevant findings (Rütsche, 2015). Informing data subjects before their data are anonymized also offers them the last chance to withdraw their data from research, which is not possible anymore once any link between them and their data is eliminated. 

Whereas anonymization of genetic data for secondary uses is explicitly regulated, the HRA does not provide any indications as far as non-genetic health data are concerned. In consequence, it must be assumed that, with non-genetic health data, anonymization before data reuse can be performed without the necessity to consult or even inform data subjects’ (Schweizer Bundesrat, 2009). In this case, the lawmaker has favored the interest of research over individual concerns about privacy and autonomy (Rütsche, 2015).

## Is It Appropriate to Grant Genetic Data an Exceptionally Special Status? a Reflection Based on the Swiss Experience

Switzerland represents an ideal case-study to reflect upon the implications of endorsing genetic exceptionalism for the secondary use of data. Indeed, although there have been some calls for considering the implementation of regulation granting genetic data an exceptionally special status with respect to secondary use requirements for research (McGuire et al., 2008), to our best knowledge the Swiss legal system is unique in having fully endorsed this stance. Moreover, Switzerland is one of the many countries that is striving to develop a learning healthcare system. At a national level, there have been calls to favor those iterative processes of healthcare improvements by allowing the flow of data from care to research—and of knowledge from research to policy-making—which are the distinctive feature of learning healthcare (Boes et al., 2018). Facilitating the secondary use of data is, in this respect, a priority. In a call for research launched in 2015 by the Swiss National Science Foundation, it was emphasized that improving the conditions for data accessibility and re-usability is especially important to remedy to the underdeveloped sector of health service research (Swiss National Science Foundation, 2015).

When reuse regulation was implemented, the Swiss legislator relied on two arguments to justify the special status granted to genetic data with respect to secondary use requirements. First, it was claimed that genetic data, because of their high predictive value, contain extremely delicate personal information, whose handling—especially in the case of secondary uses—requires stricter and more demanding standards (Schweizer Bundesrat, 2009). Second, the legislator argued that genetic data, as they can reveal some of the most distinctive traits of a person, presents higher re-identification risks in comparison with other health data. In this sense, offering data subjects more control over their data before this can be shared and reused was deemed as a necessary measure to protect individuals’ privacy (Schweizer Bundesrat, 2009). These justifications are in line with the fundamental assumption of the doctrine of genetic exceptionalism, namely that genetic data are an extraordinarily sensitive type of personal information and deserves therefore an exceptionally special status (Annas et al., 1995). This assumption is mainly based on considerations about privacy, confidentiality, and security, as genetic data are deemed to have a high predictive value and to entail considerable re-identification risks (Annas et al., 1995; McGuire et al., 2008).

In our view, however, the experience of Switzerland suggests that granting genetic data a special status with respect to secondary use requirements can be problematic both from a theoretical and a practical perspective. In particular, we argue that imposing stricter reuse requirements for genetic data neglects that also other health-related data can be particularly sensitive, it overcomplicates the drafting of comprehensible consent forms, and it is not supported by empirical evidence concerning data subjects’ preferences about the reuse of their data.

### Also Non-Genetic Data Can Be Sensitive

The claim that only genetic data have a high predictive value and poses serious re-identification risks seems to be inaccurate (Rütsche, 2010), especially in the big data era. The interpretation of genetic information is, to some extent, still an infant science and, although useful, genetic prediction has not yet become as accurate as the initial hype suggested (Jostins and Barrett, 2011). Furthermore, the application of artificial-intelligence-based approaches—such as machine learning—to the medical field has demonstrated that also routinely collected non-genetic data have the potential to predict the future health status of a person (Beam and Kohane, 2018). A recent review, for example, illustrated how machine learning can be used to enhance prognostic prediction after the onset of a mental illness based on baseline neuroimaging scans (Walter et al., 2019). Moreover, although it is true that genetic information represents a key to the identity of a person (McGuire et al., 2008), it cannot be neglected that even other health-related data can easily allow the re-identification of data subjects. For example, in a study published in 2013, it was proved that it was relatively easy to re-identify individuals with a 95% confidence level starting simply from laboratory results, although these had been previously de-identified (Atreya et al., 2013). Similarly, in another study published in 2018, it was proved that also physical activity data with geographic and protected health information removed could be easily re-identified using machine learning without the need to rely on genetic data (Na et al., 2018).

The fact that also non-genetic health data can have a predictive value and can be re-identified does not entail that they are inherently equal to genetic data. Genetic data feature specific qualities, such as: 1) the fact that they provide information about family members; 2) that DNA sequence variations of an individual are unique and lifelong; and 3) that future developments of genetic risks prediction might multiply the information that genetic data provide. Our claim is rather that both genetic data and other health-related data can both be very sensitive, albeit for different reasons. For example, a medical record containing the diagnoses of a mental disease or whether the patient is HIV positive are both very stigmatizing details about a data subject, even if they would not fall under the category of genetic data. As it has been argued, "it is not always clear what intrinsic properties of the DNA molecule (e.g., DNA sequence, genetic mutation) make it more deserving of protection than other types of information contained in the medical record of an asymptomatic, at-risk person (e.g., familial history of disease, cholesterol level, and high blood pressure)" (Dupras et al.,2018:2). On the same line, the National Committee on Vital and Health Statistics—an advisory body of the United States Federal government for matters concerning health data and privacy—repeatedly recommended to consider several categories of data as sensitive, including not only genetic data, but also mental health information, data about reproductive health and substance abuse (National Committee on Vital and Health Statistics, 2008; National Committee on Vital and Health Statistics, 2010). All these categories of data concern intimate aspects of people’s lives and could be similarly be misused for discriminatory purposes.

Therefore, from the perspective of data subjects’ privacy, it would seem more appropriate that a distinction (if any) in regulatory requirements for secondary use were based on the degree of sensitiveness of personal data, or simply on the degree of de-identification, rather than on the genetic or non-genetic nature of the data themselves. As it has been argued, the fact that genetic data are qualitatively different does not *per se* justify exceptional protection, since *all* data subjects’ information deserves in principle privacy protection (Sulmasy, 2015). The type and nature of the data is undoubtedly an important element to determine whether special protection should be granted. However, perception about the sensitiveness of data might also be influenced by elements such as: 1) whether the data are shared and reused cross border; 2) whether the data were initially collected under a strong assumption of confidentiality (e.g. medical history or notes taken during a psychotherapy); 3) what conditions there are for allowing reuse by third parties, especially industry.

If it offers special protection to certain categories of personal data only depending on the nature of the data and not on the context or the level of de-identification, regulation might secure legal certainty, but also produce counterintuitive consequences. For example, the GDPR offers special protection (GDPR, 2018, art. 9) to health data in general regardless of the context, which implies that the mere information that a person carries glasses would receive special protection with respect to data processing (Paal and Pauly, 2018). On the contrary, data about the economic relationships would not be offered the same protection, despite being arguably quite more sensitive than the information whether one carries glasses (Paal and Pauly, 2018). Moreover, relying only on the nature of the data to determine sensitiveness has the further drawback that the definitions of different categories of data provided by the law are often quite generic and open ended, and might not correspond to the complexity of current data rich research.

### Different Rules for the *Default Option* Hinder the Creation of Clear Consent Forms

**Figure 1:**
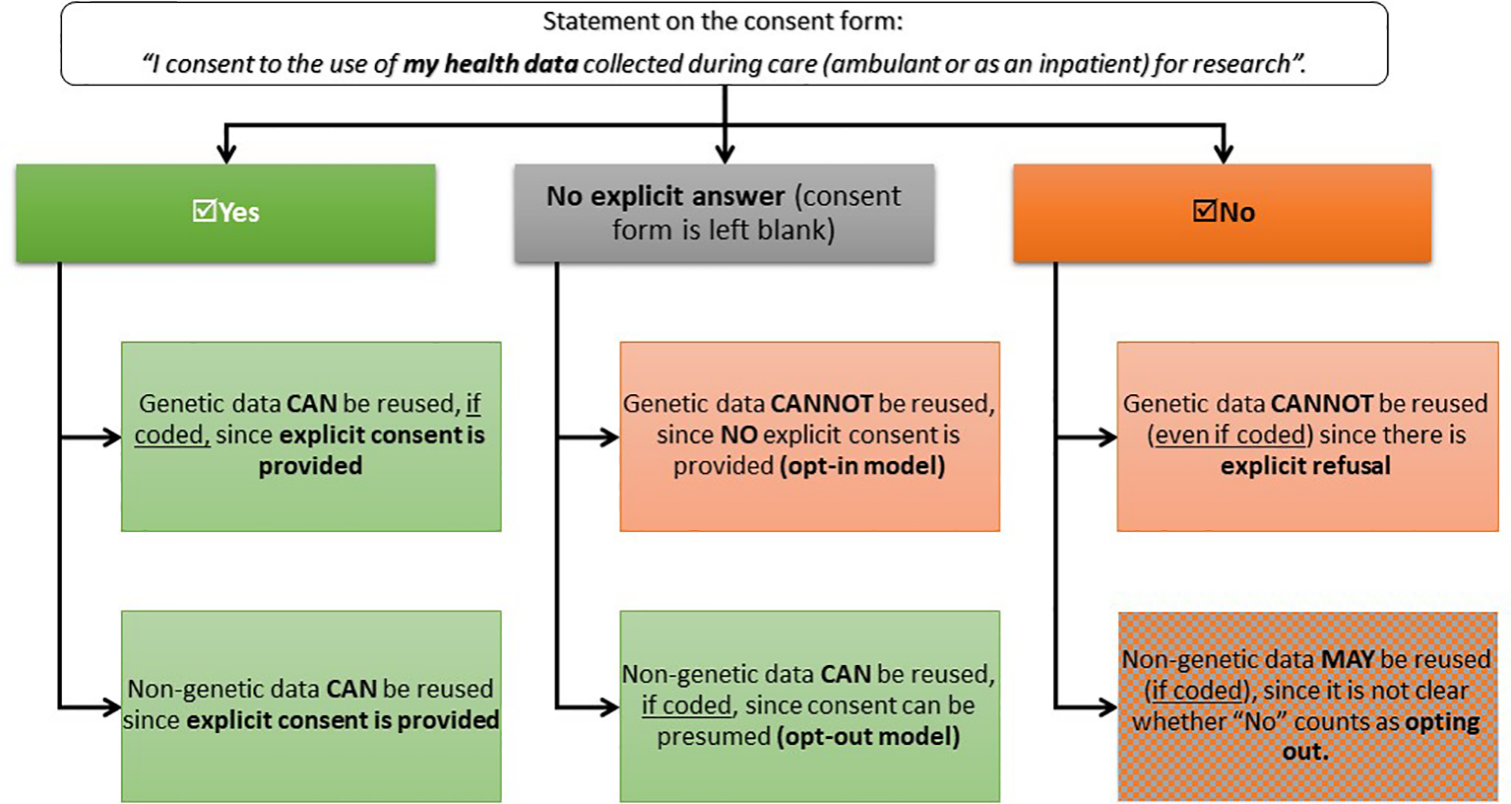
Legal consequences of data subjects’ replies on consent forms in Switzerland. This decision-tree illustrates what are the consequences of the different choices that data subjects can take when compiling the consent form. Under the current national policy, patients that enter a care facility (ambulatory or hospital) should be provided with a consent form that explains them how their data collected during clinical care might be reused for research and then asks them whether they would consent to the secondary use of their data.

Secondly, the presence of different regulatory requirements complicates the process of drafting clear and reader-friendly consent forms, thus impacting on transparency and trust in the researchers. In fact, the presence of different legal standards for the two types of data entails that a consent form should ensure that data subjects understand: 1) the difference between genetic and non-genetic health data, and 2) the different consequences that stem from not providing explicit consent. As to the first point, whereas the distinction between genetic and non-genetic data can be clear-cut from the perspective of researchers, the same does not necessarily hold true for lay people. A solution could be the elaboration of tiered consent forms, where the data subject can elicit the types of studies that they want their data to be reused for (Bunnik et al., 2013). But even if tiered consent forms were to be used, the problem would remain that patients would need to understand how the same answer on the consent form has different implications depending on the type of data it refers to, because of the diverging regulatory standards. In Switzerland, for example, *explicit* consent is required for the reuse of genetic data (opt-in model), whereas non-genetic health data can be used even without explicit consent if they are "coded," unless the subject explicitly opts out (opt-out model). This entails that the *default option* (i.e. what happens if no explicit answer is provided) for the two types of data is different, thus causing some divergence in the consequences that the same answer in the consent form has for genetic or non-genetic health data (see **Figure 1**).

Indeed, in Switzerland the creation of an appropriate consent form following the regulatory requirements for the reuse of genetic and non-genetic data has proven to be a challenge. Since 2015 the Swiss Academy for Medical Sciences, Swissethics (the umbrella organization of all regional RECs, also responsible for coordinating and harmonising the ethical overview of research), and Unimedswiss (an organization of all university hospitals) have been attempting to elaborate an appropriate consent form that would mirror regulatory standards. A first draft was published in 2017 (Swiss Academy of Medical Sciences, 2017), but it was soon criticized by patient organizations, single hospitals and representatives from the research community. These denounced the lack of clarity in terms and formulations and described as potentially deceptive the procedure of how consent for the reuse of non-genetic data in a “coded” form could be implied as long as the subject does not explicitly dissent (Swiss Biobanking Platform, 2018), which is a consequence of having different regulatory requirements for this kind of data. A study put this consent form to the test with some subjects and confirmed that the difference in regulatory standards between genetic and non-genetic data can be difficult to convey (De Nardi et al., 2018). The study concluded that “the fact that different levels of data protection—depending on the type of data (genetic vs. non genetic)—are legally stipulated creates a potential problem of comprehension” (De Nardi et al., 2018:27).^5^ A subsequent version for a uniform consent form to be used throughout the country was drafted as a reaction to these criticisms and it *de facto* abandoned any significant distinctions between genetic and non-genetic data, by requesting data subjects to *explicitly* consent or *explicitly* dissent for the reuse of both (Unimedswiss, 2019). This confirms that, although setting more relaxed regulatory standards for non-genetic health data is aimed at facilitating their reuse, this objective might backfire. Having different *default options* for genetic and non-genetic data comes at the price of reducing the clarity of consent forms, which is key to promote data subjects’ support and participation rates, especially for those research projects aimed at improving clinical care.

### Empirical Evidence Suggests Data Subjects Do Not Support Genetic Exceptionalism

Setting different regulatory requirements for genetic data also seems to go against the findings of empirical research investigating data subjects’ preferences concerning the secondary use of data in different countries. In the United States, a conjoint analysis study with 3064 participants exploring public preferences about reuse of electronic health information found that the nature of the data does not affect subjects’ willingness to agree to the reuse of their data (Grande et al., 2013). According to this study, subjects’ concerns about secondary use of their data refer to the purpose (e.g. marketing, quality improvement, research) of data reuse, rather than to the genetic or non-genetic nature of data. Despite inherent limitations of their study, the authors explicitly conclude that their “finding contrasts with the notion that patients view genetic information as particularly sensitive” and that “it may add support to the arguments against privileging genetic information, as some experts have argued” (Grande et al., 2013:1802). Another quantitative study with a sample of 2945 participants was conducted in the Unites States with the objective of exploring cancer patients’ views concerning the secondary use of their health information (Grande et al., 2015). Even this study concluded that “although policymakers, clinicians, and ethicists tend to add extra protections to genetic information because of concerns over reidentification, discrimination, and the unknown significance of certain findings based on current knowledge, the cancer participants in our study were more willing to share their information when inherited genetic results were included” (Grande et al., 2015: 381). The authors hypothesise that cancer patients might be further motivated to allow the reuse of their genetic data since they realise the importance of this type of information and the benefit it might bring to society and research. A qualitative study from the United States on the views of prospective participants in research concerning data sharing went even further and it explicitly concluded that data subjects often see non-genetic medical data as more sensitive than genetic medical data (Trinidad et al., 2010). In this study, many of these prospective participants argued that non-genetic health data are often shared with healthcare providers under the assumption that it will be treated with confidentiality, and should thus be considered even more sensitive than genetic data. Prospective participants were particularly worried about the potentially stigmatizing contents of their confidentially shared non-genetic medical records, e.g. concerning their reproductive or mental health. As far as Switzerland is concerned, a qualitative study with semi-structured interviews was conducted in 2017 to investigate the attitudes of older adults towards the sharing of genetic data (Mählmann et al., 2017). In this case, participants were split: half of them considered that the two types of data should be treated differently; the other half expressed their opposition to any differentiations between genetic and non-genetic data. Interestingly, those who were in favor of no differentiation justified such belief by mentioning the conviction that both types are equally important for the progress of medical knowledge. In general, the great majority of the participants to this study underlined their confidence that making genetic data available for research was important to contribute to the common good and to the acceleration of research.

Although not conclusive, all this empirical evidence suggests that, when it comes to secondary uses, data subjects do not feel strongly about the formulation of exceptional protection for genetic data. On the contrary, the public seem to agree that even non-genetic data should be treated as sensitive and they reveal awareness as to the importance of making genetic data available for research. Such positive attitude by the public towards research with genetic data might not be sufficient to justify an opposition to genetic exceptionalism "in general," but it provides convincing evidence against the adoption of genetic exceptionalism in regulation concerning the secondary use of data for research.

## Actionable Recommendations and Conclusions

Designing a supportive normative framework for the reuse of data is of crucial importance for the development of a successful interaction of research and clinical care. The example of Switzerland suggests that granting genetic data an exceptionally special status does not provide a satisfactory policy choice to this aim. In Switzerland, the distinction between genetic and non-genetic health data represented an attempt to strike a balance between the interest of research in having easy access to individual data and the protection of personal privacy and autonomy. However, implementing genetic exceptionalism resulted in a multi-level regulation that is a barrier to the free flow of data between care and research without a convincing justification. Moreover, the complex Swiss reuse regulatory framework negatively impacts on normative clarity, thus not only hindering healthcare research, but also compromising individuals’ understanding and control over their personal data.

Differences with respect to secondary use standards between states can already be a significant obstacle for research (Mittelstadt and Floridi, 2016) and creating further differentiation in terms of reuse requirements within a single legal system adds to this problem. Although genetic data are undoubtedly sensitive, it is also true that medical information as a whole is highly private, valuable and requires appropriate safeguards (Evans and Burke, 2008). For the context of Switzerland, this was confirmed by a recent study conducted with Swiss RECs and exploring the attitudes towards research with human tissues, where it emerged that REC members considered clinical data in general—and not genetic data in particular—as an element whose presence required stricter consent requirements (Colledge et al., 2018). If any distinctions in terms of secondary use requirements were to be present, they should thus be based on the sensitiveness of information, rather than simply on its genetic or non-genetic nature.

In today’s healthcare systems, it is crucial to strike the correct balance between protection of personal information and facilitation of data reuse for research purposes. Although this is no easy task, it is important that regulation is confronted with those practical issues that it raises and that it is note based on purely normative claims. In this sense, setting higher regulatory standards for genetic data cannot come at the price of the law being too articulated and potentially disorientating for both research institutions and data subjects. In Switzerland, dissatisfaction with the current regulatory framework has been voiced also by Swissethics and, in a recently delivered report, it has been suggested that the special status granted to genetic data is one of the most problematic aspects the legislator should revise (Swissethics, 2018). Indeed, the whole Swiss regulation concerning human research is currently under evaluation by the Federal Office of Health, and one of the key points of such evaluation concerns exactly the question whether rules concerning secondary use are appropriate (Bundesamt für Gesundheit, 2017). For this reason, it is to be hoped that Switzerland will soon align with other national and international regulations and that, with respect to secondary use requirements for research, genetic data will continue to be considered important, but not exceptional. Thereby, we do not argue that reuse requirements for genetic data should necessarily be more relaxed, but simply that legal standards should not differ between genetic and non-genetic data. Whether requirements are strict or relaxed is something that depends on the cultural and societal circumstances where legislation is enacted. In fact, any regulatory and procedural burdens have their *raison d’être*, but only when they have a good justification and the alternatives are worse. In the case of secondary use, different standards between genetic and non-genetic do not seem to be justified, since they neglect that also non-genetic health data can be very sensitive, and they are not the best alternative, since having the same standards—whether strict or relaxed—for all kinds of data would simplify the consent process, help secure the trust of data subjects and ensure that reuse of already collected data is rightfully promoted.

## Author Contributions

AM, BE, and SM conceived the initial idea of the paper. AM prepared the initial draft under the guidance of SM and BE, who continuously provided feedback and comments. LG and CP-M reviewed the draft, provided feedback about the regulation analysis, brought in additional motivations against genetic exceptionalism, and finalized the discussion parts. All authors read and approved the final version of the paper.

## Funding 

This work was supported by the Swiss National Science Foundation (SNF NRP-74 Smarter Health Care, grant number 407440_167356). The funder had no role in the drafting of this manuscript and the views expressed therein are those of the authors and not necessarily those of the funder.

## Conflict of Interest

The authors declare that the research was conducted in the absence of any commercial or financial relationships that could be construed as a potential conflict of interest.

## Footnotes

1 By this term we refer to medicine which “aims to prevent, diagnose and treat diseases by taking into account individual variability of genes, environment and lifestyle for each person” (Meier-Abt et al., 2018).

2 There are some cases where biologic material has been reused for a high number of times. The most notable one is that of Henrietta Lacks (Lasso, 2011)

3 The research exemption has been given a broad scope of application by the GDPR and should facilitate the performance of research without the need to obtain consent (Shabani and Borry, 2018). However, the GDPR also presents open clauses with respect to the processing of data for research purposes. Article 9(2)j and article 89 list “research” as a legitimate ground for the processing of data and delegate to member state the possibility to define on which further terms. This openness has been used, for example, by Denmark in art. 10 of the new Data Protection Act enacted at the national level to supplement the GDPR.

4 Naturally, it remains a challenge to define to what extent genetic data can truly be de-identified. In fact, although it might be difficult to re-identify data subjects from single portions of their DNA, the whole genome data of a person is so unique that it is always possible to track back the data subject. However, whether data can be considered as “de-identified” or “anonymized” depends also on the legal definition of de-identification or “anonymization.” The GDPR, for example, does not directly define anonymization, but explains that “the principles of data protection should therefore not apply to anonymous information, namely information which does not relate to an identified or identifiable natural person or to personal data rendered anonymous in such a manner that the data subject is not or no longer identifiable” (GDPR recital 26). It then adds that “to determine whether a natural person is identifiable, account should be taken of all the means reasonably likely to be used, such as singling out, either by the controller or by another person to identify the natural person directly or indirectly. To ascertain whether means are reasonably likely to be used to identify the natural person, account should be taken of all objective factors, such as the costs of and the amount of time required for identification, taking into consideration the available technology at the time of the processing and technological developments” (GDPR recital 26). From a more technical perspective, “de-identification” or “anonymization” is defined as the process of eliminating all direct identifiers and indirect (or quasi) identifiers, i.e. the metadata related to the information collected from a data subject (i.e. name, social security number, date of birth etc.) (El Emam et al., 2015).

5 Translation from the original text in German. The original reads “die Tatsache, dass unterschiedliche Stufen des Datenschutzes—je nach Art der Daten (genetisch vs. nicht-genetisch)—gesetzlich vorgeschrieben sind, stellt ein potenzielles Verstehensproblem dar”.

## Abbreviations

GDPR, General Data Protection Regulation; HIPAA, Health Insurance Portability and Accountability Act; TCPS 2, *Tri-Council Policy Statement 2: Ethical Conduct* for *Research Involving Humans;* PIPEDA, Personal Information Protection and Electronic Documents Act; HRA, Human Research Act; HRO, Human Research Ordinance; REC, research ethics committee; IRB, Institutional Review Board; SAMS, Swiss Academy of Medial Sciences.
